# Design and evaluation of a web-based electronic health record for amblyopia

**DOI:** 10.3389/fmed.2024.1322821

**Published:** 2024-04-04

**Authors:** Roya Naemi, Mina Akbarian, Maryam Ebrahimi, Leila Shahmoradi, Babak Masoomian, Sorayya Rezayi

**Affiliations:** ^1^Department of Health Information Management, School of Paramedical Sciences, Ardabil University of Medical Sciences, Ardabil, Iran; ^2^Department of Health Information Management and Medical Informatics, Tehran University of Medical Sciences, Tehran, Iran; ^3^Department of Health Information Technology, Neyshabur University of Medical Sciences, Neyshabur, Iran; ^4^Department of Health Information Management and Medical Informatics, School of Allied Medical Sciences, Tehran University of Medical Sciences, Tehran, Iran; ^5^Farabi Eye Hospital, Tehran University of Medical Sciences, Tehran, Iran

**Keywords:** amblyopia, electronic health record, design, evaluation, usability test evaluation

## Abstract

**Introduction:**

Amblyopia, or lazy eye, is a type of visual impairment in which the eyesight is not complete, even with the use of glasses. For the treatment of this disease, accurate and continuous examinations are needed. Nowadays, patient-centered care, by relying on web-based electronic records for amblyopia, has the potential to reduce treatment costs, increase the quality of care, and improve the safety and effectiveness of treatment. Therefore, the purpose of this study is to design and evaluate an Electronic Health Record (EHR) for patients with amblyopia.

**Methods:**

The present study is applied developmental research. Using a Morgan table as a sampling tool, a straightforward random sampling technique selected 150 records from 1,500 records that were free of flaws. The design of the electronic version proceeded in a cascading manner so that after the design of each part, it was presented to the amblyopia experts, and if approved, the next part was designed. To design this EHR, the C# programming language and MySQL database were used. A system evaluation was performed by entering and recording patient information. For this purpose, the standard Questionnaire of User Interaction Satisfaction (QUIS), consisting of 18 questions, was used.

**Results:**

According to the amblyopia EHR data elements, the data of physician and patient, examinations, website members, and members’ roles were determined. After defining the fields and classes that explain the tables, the EHR was designed. The usability evaluation of the system showed that the mean selection of very good and good options by the users of EHRs was over 90%, indicating the patients’ acceptance of web-based EHRs.

**Conclusion:**

The design of an EHR for amblyopia is an effective step toward integrating and improving the information management of these patients. It will also enable the storage and retrieval of patients’ information to reduce and facilitate the control of amblyopia complications.

## 1 Background

Eyes and sense of sight are very important in the perception of the environment, and any eye problem will have social and psychological consequences, so taking care of the eyes and paying attention to diseases that affect the eyes’ health are vitally important (1, 2). Amblyopia, or lazy eye, is a type of vision disorder that causes a decrease in central vision in an apparently healthy eye (3). In this case, although the external structure of the eye is healthy, the vision is not perfect, even with the use of glasses. When the development of vision in one eye is normal and abnormal in the other eye, the eye with poor vision becomes lazy (4). The most common cause of amblyopia is strabismus, and the less common cause is anisometropia (refraction difference between two eyes). It may also be caused by a combination of strabismus, anisometropia, or visual deprivation (5). Although the pathophysiology of amblyopia is not fully known, regardless of the type of amblyopia, many ophthalmologists have recommended that its treatment should be started as soon as possible (6). Improvement of vision after treatment has a good probability of success only at the age of less than 6 years.

In studies conducted in Iranian universities, the prevalence of amblyopia has been reported between 1.03 and 1.2 (7, 8). With the prevalence of this amount of eye laziness and the importance of its treatment, the need for a database to record the information about this disease is felt, and today hospital management information systems are considered one of these tools in the health sector (9). On the one hand, the need for these systems has raised due to the increasing complexity of processes and information in the health field, and on the other hand, their supply has been combined with significant diversity and innovations (10, 11).

In recent years, investment and implementation of information technology application plans in the health sector have been the main priorities of advanced countries (9, 12, 13), and this importance has also been emphasized by the World Health Organization (WHO) (14). It can be concluded that treatment and follow-up of any disease require storage and retrieval of information (15), so the use of efficient information systems to improve the efficiency, effectiveness, and quality of services and customer satisfaction is an undeniable necessity (16–19). One way to access information in the best possible way is to use an Electronic Health Record (EHR). The EHR is an important tool for providing high-quality care through the sharing of health information. The benefits of EHR increase when patient-recorded information is available and used by all those involved in patient care (20, 21). Healthcare providers electronically record, verify, and share patients’ information in the EHR system. The important administrative clinical data pertinent to the patient’s care under a specific provider is included in this system. This data includes demographics, progress notes, problems, medications, vital signs, past medical history, vaccinations, laboratory results, radiology reports, genetic and phototype information, and more (21). Timely access to reliable information improves the quality of service delivery and treatment processes and speeds up decision-making, implementation of preventive activities, monitoring of the disease process, early detection of complications, and timely treatment (22, 23). Paper-oriented records are not reliable sources of health information due to their inherent limitations, such as handwriting errors, misspelled words, increased costs of printing, and the need for a large volume of folders, archives, and specialized manpower (24).

The design and use of a web-based EHR for patients leads to integrated data collection, prevention of information loss and dispersion, illegibility of written instructions, reduction of unnecessary actions, as well as improvement of workflow, efficiency, and reduction of medicinal costs (including reduction of medical errors, drug side effects, and so forth.) (24–27). Additionally, the use of EHRs is crucial for the treatment of illnesses, particularly those like amblyopia for which there is no known cure. This is because improved access to patient medical records allows medical staff to diagnose patients more accurately, leading to the provision of more appropriate treatment. They also avoid repeated and pointless tests and prescriptions and are informed about the patient’s records by the physicians. The price of tests and medications can be greatly reduced by using EHR system. The use of digital and smart technologies has greatly advanced case design in recent years. A blockchain-based EHR system can be developed and validated as part of clinical projects. The purpose of such systems is to enhance medical record storage and enable provider-to-provider data sharing. They also aimed to reduce environmental uncertainty (28).

Healthcare professionals are primarily responsible for compiling data within an EHR, and only specialists have exclusive access. Furthermore, it is important to note that only a single healthcare practitioner has the capability to furnish information for an EHR. A novel software application, referred to as a Centralized Electronic Medical Record System (CEMRS), has been developed. The primary objective of this program is to offer a comprehensive and secure EHR system, facilitating healthcare providers access to patient medical records at any given time and from any location (29). Effective, efficient and satisfactory use of the system to perform assigned tasks is called EHR usability (30). Studies indicate that the use of EHR systems will be associated with the poor ability of adverse results in the provision of health services, including low participation, job dissatisfaction, medication errors, mortality, and readmission (30). Increasing safety, appropriately managing patients, and enhancing cognitive function are all correlated with better system usability (31–33). The usability and beneficial implementation of the product in the complex and real-world clinical environment are fostered by the user-centered design process of the EHR and the evaluation of system usability during design and implementation (34). It has been suggested that ways to improve the system’s added value and make the EHR more user-friendly include standardizing notes, employing reminders in place of memory, creating a dashboard to track patients’ treatment progress and connect relevant data, and automatically computing information (35). Considering that so far, no research has been done in Iran to design and evaluate a web-based EHR for amblyopia, the researchers in this study aimed to design and evaluate this system in Farabi Hospital of Tehran University of Medical Sciences to provide quality services, prevent the progression of this disease and introduce an important resource for education and research.

## 2 Materials and methods

The current investigation is classified as an applied-developmental study that was conducted in two distinct stages. The initial phase encompassed the actual design of the web-based EHR using the extracted Minimum Data Set (MDS). Determining the MDS needed to design the web-based EHR is performed in the previous work of the authors (36). The second phase entailed evaluating the effectiveness and functionality of the designed web-based EHR for amblyopia. The principal two phases of this research are presented in the following:

### 2.1 Designing the web-based EHR

By studying the clinical records of patients with amblyopia in Farabi Hospital and related articles, software, systems, and electronic records, a web-based EHR for patients with amblyopia was designed. Samples were taken from the Amblyopia Clinic of Farabi Hospital. The main elements to be completed in the EHR were identified in the previous study (36). The design of the electronic version proceeded in a cascading manner so that after the design of each part, it was presented to the amblyopia experts, and if approved, the next part was designed. Corrections were applied to the electronic version of the system. In the design phase of the web-based EHR for amblyopia patients, MS SQL Server software, ASP.NET MVC web programming language, and C# basic programming language were used to design the database tables and manage the system database. HTML5, JQuery, and CSS technologies were also used to design the appearance and appeal of the pages. Entity Framework was used for database connections, and IIS Virtual Server was utilized for setting up, running, and testing the system. Also, since the EHR for patients with amblyopia was designed to be web-based, there was a need for the researcher to purchase a dedicated domain from hosting companies.

### 2.2 Evaluating the web-based EHR

A descriptive-analytical study was used in the evaluation phase. The research population during the evaluation phase included the amblyopia experts and the fellowships, residents, and nurses of the Amblyopia Clinic of Farabi Hospital, whose opinions were used through a questionnaire. At this stage, a strategy similar to experimental conversion was applied, and the EHR was used experimentally in the Amblyopia Clinic of Farabi Hospital. A Morgan table was used for sampling and 150 records without defects were selected from 1,500 records by a simple random sampling approach. Then, the information of patients with amblyopia was entered into the web-based EHR and its software and outputs were evaluated. The QUIS standard questionnaire was used to assess the system’s usability. In other studies, the reliability and face validity of the Persian questionnaire have been confirmed. This questionnaire was customized by the research team in this work (37, 38). Notably, we adjusted the items of the chosen standard questionnaire to increase the cooperation of the recruited physicians (See Supplementary Appendix Table A1). This modified questionnaire consists of 18 questions in five sections. The first section includes five questions related to the general use of the system; the second section, with three questions, is related to the display screen; and the third section, with three questions, is related to the system terminology and information. The fourth section, with four questions, is related to EHR learning capability, and the fifth section, with three questions, is related to the overall capabilities of the EHR. To answer the question, five options—very good, good, average, bad, and very bad—are considered. The sampling method was a census, and there are 20 ophthalmology residents, fellowships, specialists (professors), and nurses of the Amblyopia Clinic of Farabi Hospital who are included in the sample of this research. After distributing 20 questionnaires among the research community, their responses to the questionnaire and the results of the questionnaire were analyzed. The participants who took part in this evaluation included four amblyopia specialists, three fellowships, seven ophthalmology residents, and six nurses working at Farabi Hospital.

## 3 Results

To answer the question of designing and evaluating a web-based EHR for amblyopia and achieving the research objectives, the findings were divided into four main parts:

### 3.1 Database table design

**Figure 1:**
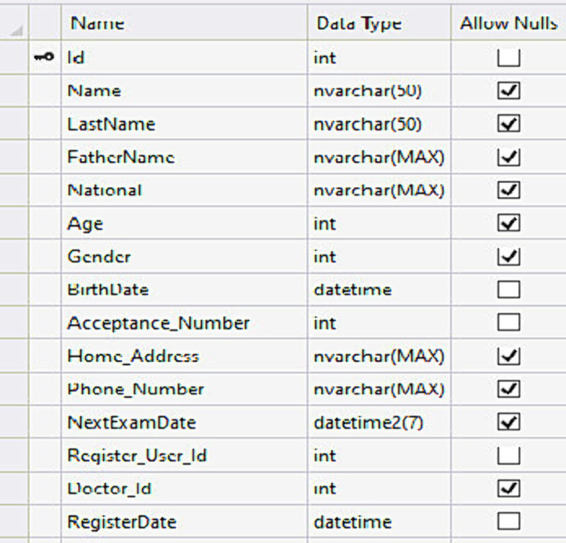
Patient information table.

In this step, according to the findings of the first step (determining the MDS required to design the electronic record of amblyopia patients on the web), the fields of the electronic record database tables were determined. The distribution of table fields was done according to the needs of system users and the existence of connections between fields. The desired tables include the patient information table, the attached record information table, the examination information table, the site member information table, the role definition table, and the role assignment table for members. An example of a patient information table is shown in Figure 1.

### 3.2 Final design with related codes

At this stage, according to the amblyopia EHR data elements, the data of physician and patient, examinations, website members, and members’ roles were determined. After defining the fields and classes that explain the tables, the database was created. The “Entity Framework Code First” was used to create and work with the database in this project. In this method, tables are defined as classes and then a database can be created and manipulated by creating a context class. Each table in the database requires a class, and these classes are located in the model folder in the Doctor Model section. The first class was the patients’ table creation class. In this class, the table fields were defined as properties, and since each patient could have multiple examinations, the relationship between this class and the examination class was defined as Virtual & ICollection. The examination class contained the data of examinations performed by physicians. This table had a one-to-one ratio with the patient and physician tables and a one-to-many ratio with the attachments table. The next class was the physician class, which is used to store the information of website users. To determine the table’s primary key, the Annoticate was used as the key. The examination field was expressed as a set. This means that each physician is able to register several examinations in own name. Attachments are also recorded in the attachments table, the definition class of which is as follows:

**Pseudo code for the class definition**

[Table ("Attachment")]

Public partial class Attachment

[Key, DatabaseGenerated (DatabaseGeneratedOption.Identity)]

Public int f_id {get; set;}

Public string FileName {get; set;}

Public string date {get; set;}

Public string FilePath {get; set;}

Public int? Size {get; set;}

Public virtual Exam exam {get; set;}

The first part of the codes of the site pages will examine the pages related to membership and the entry and exit of site members. In the previous section, a separate class was defined for each of the database tables. These classes were only useful for creating the database and accessing the fields of the database tables. Now the desired model classes must be rewritten. With this, error control can be placed on the fields of the Annoticate class. In the AccountViewModel class located in the Model folder, the desired class for the membership section is as follows:

**Pseudo code for the registration page**

public class RegisterViewModel

{

[Required]

[Display(Name = "UserName")]

public string UserName {get; set;}

[Required]

[StringLength(100, ErrorMessage = "The {0} must be at least {2} characters long.", MinimumLength = 6)]

[DataType(DataType.Password)]

[Display(Name = "Password")]

public string Password {get; set;}

[DataType(DataType.Password)]

[Display(Name = "Confirm password")]

[Compare("Password", ErrorMessage = "The password and confirmation password do not match.")]

public string ConfirmPassword {get; set;}

public string Name {get; set;}

public string Family {get; set;}

### 3.3 User interface of designed EHR

**Figure 2:**
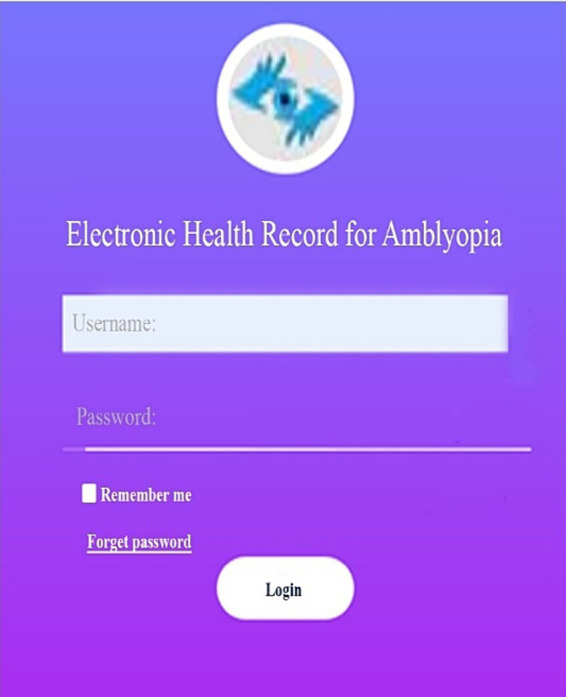
The login page.

By entering the address http://ambly.hisapps.ir, you can access the electronic record of amblyopia patients on the web. By entering this address, the first page viewed is the login page, which can be seen in the middle of the main page, with the password and password sections. If a password and username have already been defined for the user, he can access the electronic record by entering them (Figure 2).

The program menu is in the form of a black bar in the Persian part on the right side and in the English part on the left side, which includes sections such as physicians, patient lists, new patient registration, user and management, which includes all users and creating a new user, clinical information, sensory tests, physicians’ prescriptions, treatment plans, reports, and charts.

**Figure 3:**
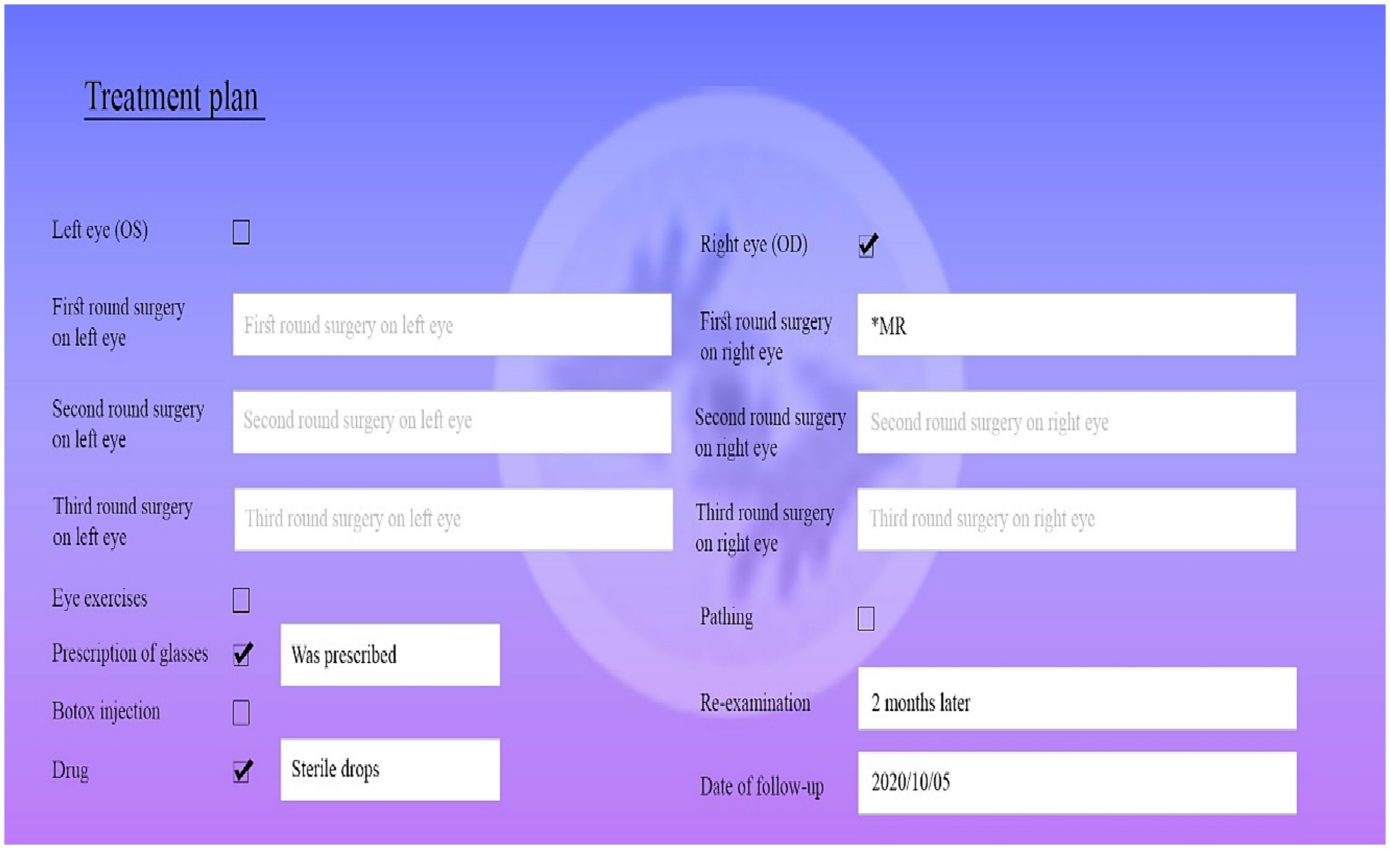
The page of treatment plan.

In the search section, you can access the history of the disease, the history of eye diseases, the history of drug use, the history of eye surgery, the patient’s main complaint, the time of the disease’s onset, and the causes of the disease. The section on visual examinations includes examinations with glasses, examinations without glasses, refraction of the eye with eye drops, and refraction of the eye without eye drops for both eyes, in which the eye score can be selected. The next part is the type and pattern of strabismus, which are determined after eye examinations. By specifying the following, the registration option is selected at the end and a record is created for the patient. The deviation of the eye is determined by the physician and information about the type of deviation in the patient’s eye is recorded and maintained. The treatment plan includes items such as determining surgery along with muscle type, closing the eyes (patching), eye exercises, prescribing glasses, and prescribed drugs, which are added to the patient’s record after registration. In the reports section, the number of treated patients by attending physician, the referral date and patient’s gender can be obtained. Finally, the patient’s eye photos are stored in the EHR and the physician presents treatment plan according to the patient’s issues (Figure 3).

### 3.4 Evaluation of the web-based EHR

**Figure 4:**
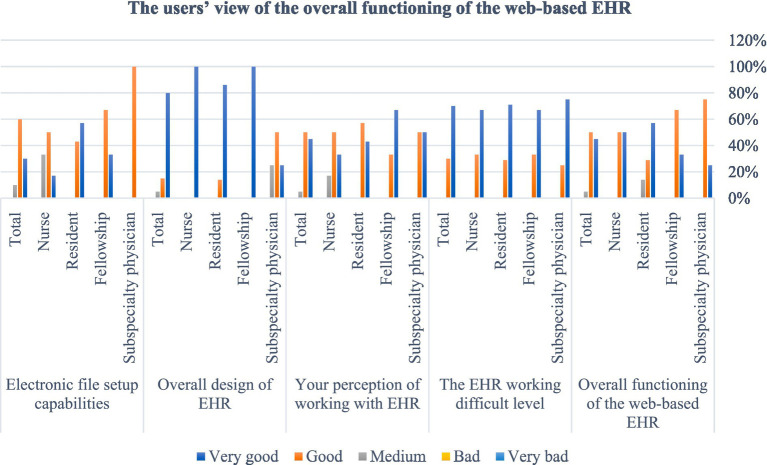
Users’ view on the overall performance of the web-based EHR for amblyopia patients.

**Figure 5:**
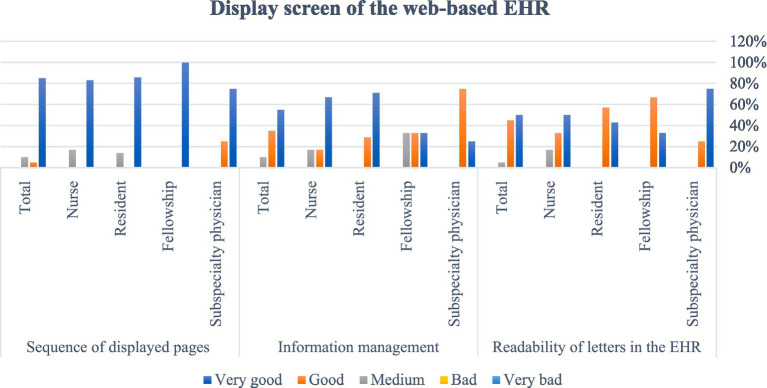
Users’ view on the display screen of web-based EHR.

**Figure 6:**
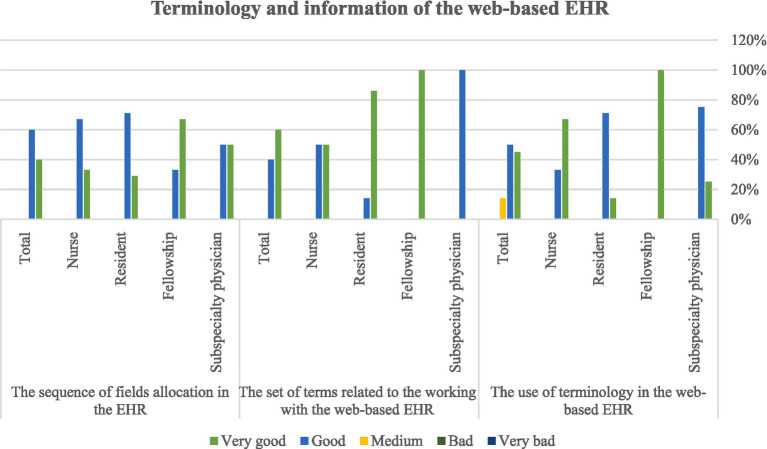
Users’ view on the terminology and information used in the web-based EHR for amblyopia patients.

After designing and programming the system, the usability and user satisfaction of the EHR were evaluated with the experimental conversion strategy. Table 1 shows the demographic information of the research community. The frequency distribution of the participant’s answers to the questions in each section is presented in separate figures. As seen in Figure 4, most of the evaluators’ comments on working with EHR were very good or good. Several physicians suggested some corrections to the latest version of the system. The second section of the questionnaire to evaluate the usability of the EHR was related to the screen capabilities of the EHR on the web of amblyopia patients, and the frequency distribution of the responses of amblyopia specialists, fellowships, residents, and nurses is presented in Figure 5. The views of subspecialty physicians, fellowships, residents, and nurses of the strabismus clinic on the terminology and information used in the web-based EHR for amblyopia are shown in Figure 6. The fourth section of the questionnaire was related to assessing EHR learning ability. Participants evaluated the usability of web-based EHR for amblyopia as very easy and efficient and finally considered it an effective tool in integrating patient information and helping to improve patient’s condition.

**Table 1:** Frequency distribution of demographic information of participants in the study.

Demographic information		Frequency	Percentage
Gender	Female	5	25%
	Male	15	75%
Scientific ranking	Subspecialty physician	4	20%
	Fellowship	3	15%
	Resident	7	35%
	Nurse	6	30%
Work history	Under 5 years	9	45%
	5–10 years	3	15%
	10–15 years	4	20%
	15 years and over	4	20%

**Figure 7:**
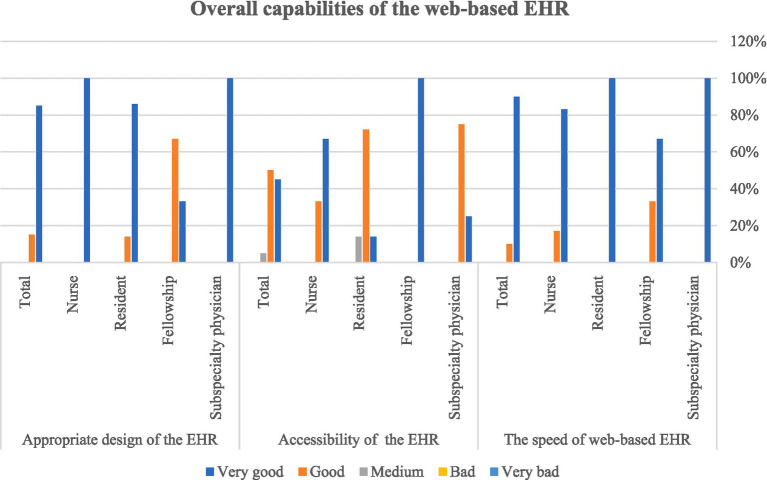
Users’ view on the overall capabilities of the web-based EHR for amblyopia.

The fifth section of the questionnaire is related to the general capabilities of the EHR and users evaluated the general capabilities of the system as very easy and efficient. The analysis of the data obtained from the questionnaire showed that the research community answered all the questions with “good” and “very good” options, and the web-based EHR for amblyopia was fully approved by the experts of the Amblyopia Clinic (Figure 7).

## 4 Discussion

In developing countries like Iran, we still face problems using EHR, and patients’ records are paper-based. Since accurate recording of patients’ information in medical records is one of the minimum requirements for providing high-quality treatment, the main goal was to design and evaluate an electronic health record for patients with amblyopia. To achieve this goal, at first, we determined the MDS, defined data characteristics in the data dictionary, defined the type and number of characters, and created structured forms for data entry that can improve the exchange of information and the quality of clinical care (36); in the following step, the designed web-based EHR is evaluated for its usability. In the present study, the web-based EHR of amblyopia patients was developed using the ASP.NET programming language and the new Visual Basic database SLOcalDB (SQL Server 2014 Express). To use the EHR, the admin of the web-based EHR defines the users in the program and provides them with a username and password, and physicians can access the EHR on the web in real time. In the web-based EHR for patients who visit the clinic for the first time, there is a page where all the patient’s demographic information, surgical records, drug use, family diseases, eye diseases, the patient’s main complaint, and the date of the patient’s first examination are recorded. After that, the examination of the patient is done according to the entered items in the EHR. Finally, the images of the patient’s eyes are stored in the EHR and the physician presents a treatment plan according to all the patient’s issues. This EHR had an easy and user-friendly interface, and users were able to easily enter the patient’s demographic and clinical information and ophthalmic images into the EHR and view this information in an integrated manner.

Updating information in the healthcare environment or settings has been possible through advanced information technology to enable continuous quality improvement in complex areas. Therefore, information can be stored, processed, searched for, and retrieved in healthcare organizations with the help of web technology (39).

Scientifically speaking, both structured and unstructured data are typically present in EHRs. Many statistical techniques can be applied with relative ease to structured data processing. Nevertheless, structured data by itself does not offer all of the details regarding the entire clinical context. On the other hand, unstructured data can offer additional, useful information, but the analytics procedures involved in using it are laborious, time-consuming, and involve a lot of manual labor. Effective use of both structured and unstructured data is necessary for a well-designed diagnosis and decision-support tool to extract valuable information and produce better results. The benefits of combining structured and unstructured data have been demonstrated by numerous studies (40). During the process of data integration and reconstruction in the EHR systems, combining structured and unstructured data can be done early or late (41).

In the present study, a web-based EHR for amblyopia was designed using the ASP.NET programming language, and a SQL Server database was also used to enable physicians and users to obtain a username and password to access the EHR. In line with our study, in the study of Ibrahim et al., the web-based ASP.NET framework was used to provide a data exchange model for patients’ records to reduce patients’ time and cost and enable physicians to obtain up-to-date and accurate information from patients’ records (39). In the study of Lee et al., ASP.NET was used to set up a platform for displaying, transferring data, accepting databases, and converting personal health record formats (42). Studies have shown that web-based EHRs allow access to and exchange of information such as patient name, history, illness, physical examination, health assessment, patient condition, quality of life, and patient care (43). Therefore, due to the web-based nature of the program and the features of the ASP.NET programming language, this language was optimal to develop a web-based EHR for amblyopia.

A large amount of heterogeneous data has posed a huge challenge to evidence-based decision-making. The use of data warehouse technology is an effective solution for aggregating and analyzing different health data. In the study of Nero et al., a data warehouse was created to investigate various factors affecting obesity. In this study, SQL Server was used to generate reports in appropriate formats to extract, convert, and load data. Analysis of health data can identify unknown links between different types of health factors and also evaluate the effectiveness of different medical methods using new data mining techniques (44).

In the present study, the SQL Server program was used to create the database and Visual Studio software was used to design the EHR. John Beazley acknowledged that the most important factor in designing an EHR is to pay attention to the user interface because it is the first place that the users encounter, and the second factor is how the users use the EHR (45). In the current study, however, the user interface was created by the researcher multiple times on paper and then presented to the users for feedback and necessary adjustments. Ultimately, the evaluation findings demonstrated that the majority of users rated the user interface as “very good” or “good.” In the area of applied, scientific, and repeatable evaluation of various stages of EHR system development, not many high-quality studies have been published. The usability of an EHR is influenced by a number of factors, such as personnel, workflow, hardware, software, organizational culture, and communication (34). Lack of formal and standard reporting of usability assessment is the main cause of knowledge gap (46). Large number of usability problems can affect effectiveness, efficiency, user satisfaction and patient safety (47). According to Joukes, the expectations and attitudes of end users during the implementation of EHR should be examined by surveys and questionnaires. The EHR usability, EHR alignment with work processes, in-service and post-implementation support, EHR training, and use of other centers’ experiences are among the important factors in EHR success (48).

A think-loud approach was used to evaluate the functionality of the HER; in this method, the EHR was provided to the users and while working with it, they expressed their opinions, and the researcher wrote down the appropriate comments and applied them to the final version. A study by Eric et al. examined the attitudes of ophthalmologists at the University of Michigan after the implementation of the EHR system. The survey questions focused on satisfaction, efficiency, and documentation of the EHR. Ophthalmologists commented on their ability to produce high-quality documents and the impact of EHR on patient interaction. They also did not report a significant change in documentation time or clinic efficiency. The results of this study determined the physicians’ areas of interest, the need for physician training, and the customization of EHR software and implementation (49). In the present study, in order to evaluate the EHR, a questionnaire was distributed among the research community and the analysis of participants’ responses showed that all participants approved the system. The participation of end users in the process of preparation and implementation of EHR helps to meet the needs of users, create a sense of ownership of participation in the project and accept the system (10). Therefore, in the present study, from the stage of determining the minimum required data to the stage of designing and evaluating the system, the opinions of the end users were examined and applied to the project. It is obvious that customizable EHR with an emphasis on users’ needs will be effective in improving patient safety. The use of technology in healthcare in the form of EHRs is considered the most important and necessary issue to foster the quality of healthcare and research has shown that it is not only a method for integrating information and representing the condition of patients and a dynamic source for health care, but also leading to access to information and clinical records, educational electronic communication and comprehensive management and ultimately improving the level of public health.

### 4.1 Strengths and limitations

The strength of our study is the survey of clinical records of patients with amblyopia in Farabi Hospital in the first stage to identify the information elements of the EHR, which led to the generalization of our work. It is noteworthy that surveys of records were conducted in one of the country’s most advanced ophthalmology centers. This study has ethical approval number IR.TUMS.SPH.REC.1398. 290 is from Tehran University of Medical Sciences and owns the registered data of Farabi Hospital in Tehran.

This study has several limitations. The evaluation of the system was conducted at Farabi Hospital with 20 ophthalmology assistants, fellowships, and specialists (professors). Overall, the study was performed in a single center with a limited number of healthcare providers. Using this system on a larger scale requires more investigations, identifying challenges, and providing solutions. Specifically, this study focused on designing a web-based EHR and evaluating its usability by healthcare professionals. The EHR was only available to users of the amblyopia department of Farabi Hospital in Tehran and was not available to all service providers. Therefore, consent was not obtained from patients to store their data on a web-based platform. End-user concerns about data security and patient and physician privacy have not been addressed about the features of the designed EHR.

Another obstacle of the study is not examining the effect of the EHR of amblyopia on the workload and fatigue levels of end users. Considering its effect on the efficiency, productivity, acceptance, and success of the system, it is necessary to study the impact of the designed system on the workload of end users and propose re-engineering of the work. It is suggested to investigate the interventional evaluation of the usability, such as designing a dashboard to observe the patient’s treatment process and link related data, using reminders instead of relying on memory, standardizing notes of the amblyopia EHR system and its impact on the workload and cognitive performance of doctors in future studies.

## 5 Conclusion

By and large, to quickly access patients’ information, eliminate the shortcomings of paper records, and improve the quality of health services, the need for a web-based EHR for patients with amblyopia is evident. EHR is one of the most important tools for monitoring the disease process and integrating and improving the information management of patients with amblyopia. The use of a web-based EHR, in addition to facilitating access to patients’ information, makes it possible to retrieve the required information to reduce and facilitate the control of amblyopia complications. The designed system has high capabilities and users can easily record and store patient information in the system. It is suggested that the web-based EHR for amblyopia patients be upgraded by designing a mobile phone educational application for amblyopia patients. The design and implementation of a management dashboard for required reports and statistics are also suggested. Furthermore, the design of EHR for other eye diseases, such as diabetic retinopathy and congenital orbital diseases, as well as self-care applications for eye diseases and remote ophthalmic systems for chronic eye diseases like amblyopia are suggested for future research.

## Data availability statement

The original contributions presented in the study are included in the article/Supplementary material, further inquiries can be directed to the corresponding authors.

## Ethics statement

The studies involving human participants were reviewed and approved by the Ethics committee of Tehran University of Medical Sciences (Ethics approval number: IR.TUMS.SPH.REC.1398.290). Verbal informed consent was obtained from the participants or legal guardians to participate in this study.

## Author contributions

RN: Data curation, Investigation, Writing – original draft, Writing – review & editing. MA: Conceptualization, Data curation, Investigation, Methodology, Software, Validation, Writing – original draft, Writing – review & editing. ME: Conceptualization, Data curation, Writing – original draft. LS: Conceptualization, Data curation, Investigation, Methodology, Writing – original draft. BM: Conceptualization, Data curation, Methodology, Supervision, Writing – review & editing. SR: Conceptualization, Investigation, Writing – original draft, Writing – review & editing.
