# Supplementary material for: Design and evaluation of a web-based electronic health record for amblyopia
Source: Front Med (Lausanne). 2024 Apr 4;11:1322821. doi: 10.3389/fmed.2024.1322821 (PMC11025453; doi:10.3389/fmed.2024.1322821)
Supplement: Supplementary file 1 [file Table_1.docx]

**Appendix**

**Table A1:** The customized items of QUIS

| Title | **Very good** | **Good** | **Medium** | **Bad** | **Very bad** |
| --- | --- | --- | --- | --- | --- |
| **Overall functioning of the web-based HER** | | | | | |
| Overall functioning of the web-based EHR |  |  |  |  |  |
| The EHR working difficult level |  |  |  |  |  |
| Your perception of working with EHR |  |  |  |  |  |
| Overall design of EHR |  |  |  |  |  |
| Electronic file setup capabilities |  |  |  |  |  |
| **Users' view on the display screen** | | | | | |
| Readability of letters in the EHR |  |  |  |  |  |
| Information management |  |  |  |  |  |
| Sequence of displayed pages |  |  |  |  |  |
| **Users' view on the terminology and information used in the web-based EHR** | | | | | |
| The use of terminology in the web-based EHR |  |  |  |  |  |
| The set of terms related to the working with the web-based HER |  |  |  |  |  |
| The sequence of fields |  |  |  |  |  |
| **Users' view on the learning capabilities of web-based HER** | | | | | |
| Learning to work with EHR |  |  |  |  |  |
| Finding the electronic file’s features through try and error |  |  |  |  |  |
| Memorizing the names and using the capabilities of EHR |  |  |  |  |  |
| Quick and easy performance of the tasks |  |  |  |  |  |
| **Users' view on the overall capabilities of the web-based EHR** | | | | | |
| The speed of web-based EHR |  |  |  |  |  |
| Accessibility of the EHR |  |  |  |  |  |
| Appropriate design of the EHR |  |  |  |  |  |
